# Supplementary material for: Long-Term Survival and Clinicopathological Implications of DNA Mismatch Repair Status in Endometrioid Endometrial Cancers in Hong Kong Chinese Women
Source: Biomedicines. 2021 Oct 4;9(10):1385. doi: 10.3390/biomedicines9101385 (PMC8533409; doi:10.3390/biomedicines9101385)
Supplement: Supplementary file 1 [file biomedicines-09-01385-s001.zip › biomedicines-1388001-supplementary.pdf]

Table S1. Antibodies and conditions used in immunohistochemistry of MMR proteins

Antibody	Source	Dilution	Antigen retrieval method
MLH1	Ventana (M1)	1:2	EDTA/microwave, 60 min, 37°C
PMS2	Centana (A16-4)	1:2	EDTA, 120 min, room temperature; Ventana Optiview amplification, 8 + 12 min
MSH2	Cell Marque (G219-1129)	1:200	EDTA/microwave, 32 min, 37°C
MSH6	Cell Marque (SP93)	1:100	EDTA/microwave, 32 min, 37°C
